# Using the RE-AIM framework to evaluate the implementation of a clinical workflow designed to identify, refer, and connect insufficiently active patients to health coaching

**DOI:** 10.1093/tbm/ibag036

**Published:** 2026-07-08

**Authors:** Garrett M Steinbrink, Jenna L Springer, Korey A Kennelty, Heather Schacht Reisinger, Britt Marcussen, Katie Imborek, Dale S Bond, Yin Wu, Lucas J Carr

**Affiliations:** Department of Health, Sport, and Human Physiology, University of Iowa, Iowa City, IA, United States; Department of Health, Sport, and Human Physiology, University of Iowa, Iowa City, IA, United States; Department of Family and Community Medicine, University of Iowa, Iowa City, IA, United States; Department of Pharmacy Practice and Science, University of Iowa, Iowa City, IA, United States; Department of General Internal Medicine, University of Iowa, Iowa City, IA, United States; Department of Family and Community Medicine, University of Iowa, Iowa City, IA, United States; Department of Family and Community Medicine, University of Iowa, Iowa City, IA, United States; Digestive Health Institute, Hartford HealthCare, Hartford, CT, United States; Digestive Health Institute, Hartford HealthCare, Hartford, CT, United States; Department of Health, Sport, and Human Physiology, University of Iowa, Iowa City, IA, United States; Department of Family and Community Medicine, University of Iowa, Iowa City, IA, United States

**Keywords:** physical activity, public health, primary care, cardiovascular disease, disease prevention, healthcare costs, implementation science, lifestyle medicine

## Abstract

**Background:**

Primary care is well-positioned to connect patients to physical activity (PA) interventions but is underutilized.

**Purpose:**

To describe the implementation of a clinical workflow designed to identify, refer, and connect insufficiently active patients to health coaching (HC).

**Methods:**

From June 2023 to March 2025, a physical inactivity screening and referral workflow was implemented in six primary care clinics within a large university healthcare system. Guided by the RE-AIM framework, descriptive analyses focused on the number and proportion of patients who (i) were screened for inactivity, (ii) were identified as insufficiently active, (iii) expressed interest in HC, (iv) were referred to HC, and (v) were connected to HC. We also assessed provider uptake, evaluated workflow modifications, and computed measures of central tendency and dispersion during the final year.

**Results:**

Monthly, a mean (SD) of 1771 (153) patients were screened. Of these, 1013 (102), or 57.1%, were insufficiently active. A median of 288 patients (29%) expressed interest in HC, of which 243 (89%) were referred. A median of 20 patients (10% of those referred) were connected to HC monthly. Among providers with patients requesting a referral, 86% referred. Workflow changes were followed by shifts in metrics, while variability remained low during the final year of evaluation.

**Conclusion:**

This workflow identifies and refers many insufficiently active patients to HC. However, there is a gap between patient HC referral and connection to support. Identifying and implementing strategies to overcome barriers and improve patient connection rates is a critical next step.

## Teaser

A physical inactivity screening and referral workflow results in many primary care patients being referred to effective behavior change support. However, the number of patients connected to support could be improved.

Implications

- **Practice:** Large healthcare systems may implement and/or modify our clinical workflow to identify insufficiently active patients and refer them to supportive physical activity programs.
- **Policy:** Federal, state, and organizational policies aimed at facilitating discussion of physical activity in primary care (e.g. increased provider reimbursement) may result in a greater number of insufficiently active patients receiving referrals to supportive physical activity programs.
- **Research:** These findings highlight a clear need to better understand the likely multi-level barriers inhibiting patients from connecting to supportive physical activity programs when referred through the primary care setting.

## Introduction

Non-communicable diseases (NCDs) impose significant health burdens individually and socially [1]. Meeting the aerobic physical activity (PA) guidelines of at least 150 min of moderate-intensity PA, 75 min of vigorous-intensity PA, or an equivalent combination of the two each week reduces the risk of developing specific NCDs, such as type 2 diabetes [2, 3], cardiovascular diseases [2, 4, 5], specific cancers [6, 7], and depressive [8, 9] and anxiety disorders [10], reducing these burdens [11]. Unfortunately, few U.S. adults meet PA recommendations [12, 13]. Given that 12% of all U.S. healthcare expenditures are attributed to physical inactivity [11], system level strategies are needed to promote PA and reduce inactivity-related health burdens.

With approximately 85% of U.S. adults—over 200 million people—attending a routine wellness visit each year [14], the primary healthcare system is well-positioned to promote PA. Unfortunately, providers face a myriad of barriers to counseling their patients on PA, including a significant lack of time, little financial reimbursement, and limited expertise and confidence in PA counseling. As such, the American College of Sports Medicine’s Exercise is Medicine (EIM)^®^ initiative recommends screening patients for physical inactivity and connecting insufficiently active patients to PA behavior change resources via referral [15]. Moreover, in 2020, the U.S. Preventive Service Task Force (USPSTF) issued a recommendation that providers refer all adult patients presenting with CVD risk factors to effective behavior change interventions, such as health coaching (HC), to improve PA and dietary behaviors [16].

Referring patients to PA resources results in small but clinically meaningful improvements in PA compared with usual care [17]. As it is well-appreciated that most of the health-promoting benefits of PA are realized when moving from little/no PA to some PA [9, 11, 18, 19], developing and optimizing systems designed to connect insufficiently active patients, in particular, to effective resources is critical to promote population health. There is therefore a need to systematically describe the impact of existing clinical workflows designed to identify, refer, and connect insufficiently active patients to supportive behavior change resources.

Guided by the Reach, Effectiveness, Adoption, Implementation, and Maintenance (RE-AIM) framework [20, 21], the purpose of this study is to describe the initial implementation of a new clinical workflow designed to identify, refer, and connect insufficiently active patients to HC. Specifically, we aim to describe: (i) the volume of patients exposed to this clinical workflow; (ii) the effectiveness of the workflow to connect insufficiently active patients to HC; (iii) changes in RE-AIM outcomes pre- and post-implementation of workflow modifications; and (iv) the stability of RE-AIM outcomes during the last six and 12 months of the evaluation period. This study will highlight the possible impacts that standardized physical inactivity screening and referral may have on patient care and population health.

## Methods

### Setting and clinical workflow

**Figure 1:**
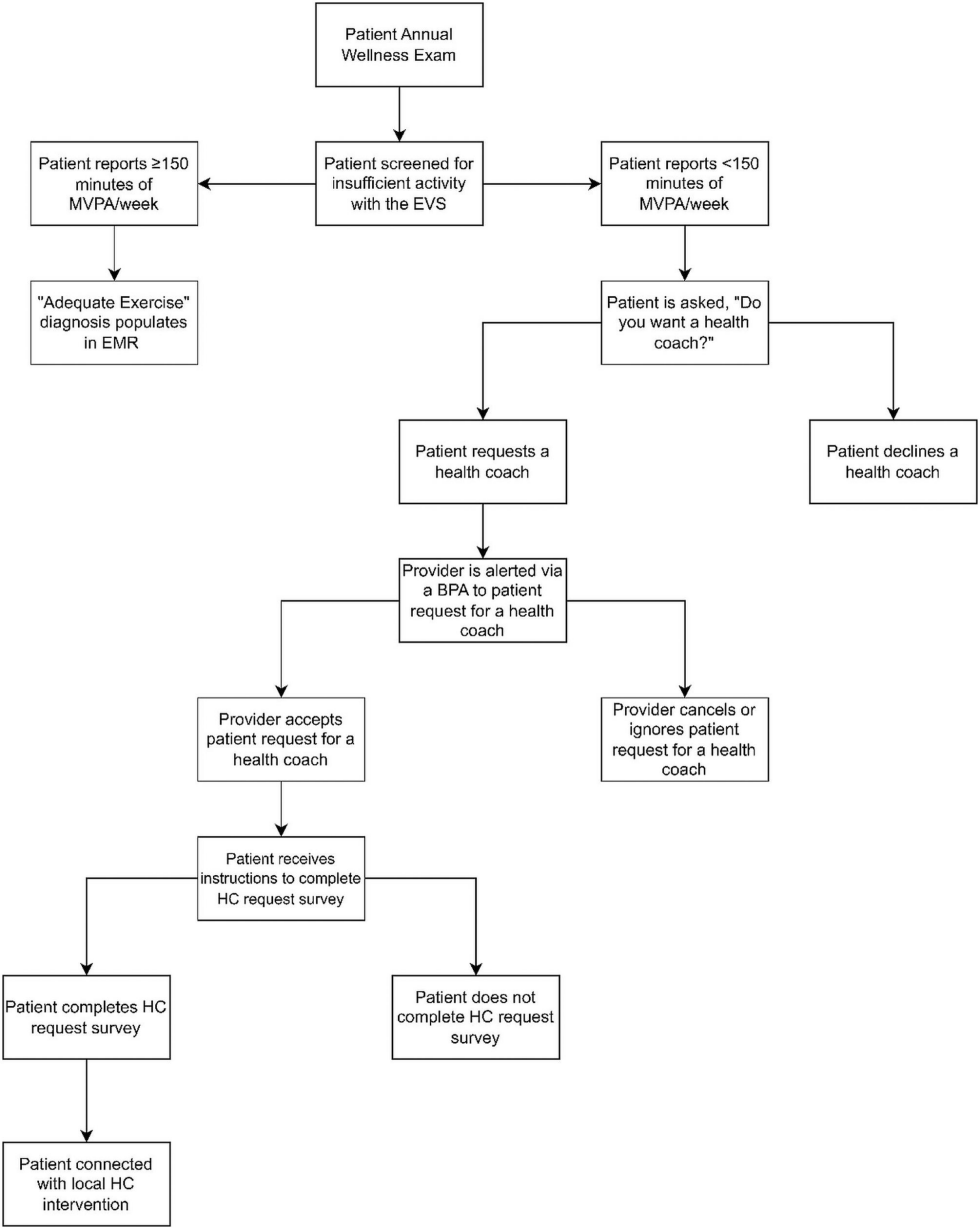
Initial clinical workflow to identify and refer insufficiently Family Medicine patients to health coaching (HC) programs.

This study was conducted in six primary care clinics within the Department of Family Medicine within a large university healthcare system. These clinics use the electronic medical record (EMR) system, Epic (Epic Systems; Verona, WI), which is widely used for patient data collection and clinical decision-making support. We have previously described our clinical workflow [22], which is shown in Fig. 1. Briefly, when new or established patients attend an annual physical exam, their aerobic PA is assessed via the two-item Exercise Vital Sign (EVS) [23]. If a patient reports <150 min of moderate- to vigorous-intensity PA (i.e. identified as insufficiently active), they are automatically asked if they are interested in meeting with a health coach. If the patient declines a health coach, the visit continues as normal. If the patient is interested in meeting with a health coach, however, their provider is automatically provided with an Our Practice Advisory (OPA) clinical decision support tool to facilitate patient referral to a local HC program within the EMR. The provider has the option to accept, cancel, or ignore the OPA. If the request is accepted, the patient receives instructions on how to connect with HC within their printed after visit summary and on their Epic MyChart mobile application (Epic Systems; Verona, WI). These instructions include a QR code and an online link to complete an HC program request form (Qualtrics, LLC; Provo, UT). Once this form is completed, the survey is automatically sent to an HC program team who contacts the patient for scheduling.

### Modifications to the clinical workflow

Led by clinic leadership, two major modifications to the clinical workflow were implemented during the 21-month evaluation period. These modifications are reported in accordance with the Framework for Reporting Adaptations and Modifications to Evidence-based Implementation Strategies (FRAME-IS) [24]. The first modification was a patient-level ‘content’ modification, changing how patients were asked about their interest in connecting with a health coach. Specifically, in month 4, the language was changed from, “*Do you want a health coach*?” to, “*Would you be interested in meeting with* a *free health coach who can help you achieve your health-related goals*?”. The goals of this modification were to: (i) ensure patients knew the HC programs were offered at no financial cost, and (ii) better clarify the role of HC programs in patient care.

A second ‘content’ modification occurred at the provider level in month 10. Specifically, prior to month 10, providers could cancel, accept, or ignore the OPA prompting a referral to HC. After observing most patient requests for a referral to HC were being ignored, clinic leadership removed the ignore option, requiring providers to either accept or cancel the OPA before closing the patient’s chart. The goal of this modification was to ensure providers were: (i) aware of their patients’ interest in meeting with a health coach, and (ii) actively participating in the clinical workflow.

### RE-AIM framework and evaluation outcomes

The evaluation of this clinical workflow is guided by the Reach, Effectiveness, Adoption, Implementation, and Maintenance (RE-AIM) evaluation framework [20]. Each RE-AIM dimension, its definition, and our specific RE-AIM outcomes are reported in Table 1.

**Table 1:** Clinical workflow outcomes guided by the RE-AIM framework, adapted from Holtrop *et al*. [20] and Stoutenberg *et al*. [21].

RE-AIM dimension and definition [20]	Exercise is Medicine® dimension definition [21]	Clinical workflow outcomes
**Reach:** The number, proportion, and representativeness of individuals who may participate in an intervention	The number, proportion, and representativeness of patients screened for PA, provided counseling on PA, and were referred to PA resources or interventions	*N* (%) of patients screened for inactivity *N* (%) of insufficiently active patients (i.e. <150 min/week of MVPA) *N* (%) of patients interested in HC *N* (%) of patients referred to HC
**Effectiveness:** The impact of an intervention on individuals, including negative outcomes, and more global outcomes (e.g. quality of life, economic outcomes, etc.)	The changes in PA, health biomarkers, incidence or risk of developing chronic diseases, and health care utilization from EIM intervention	*N* (%) of insufficiently active patients connected to HC intervention *N* (%) of patients interested in HC connected to HC *N* (%) of patients referred to HC connected to HC
**Adoption:** The number, proportion, and representativeness of individuals and/or organizations who are initiating an intervention	The number and proportion of clinics and/or individual health care providers who integrate one or multiple components of the EIM clinical workflow (i.e. screening, counseling, and referral)	*N* (%) of providers with patients interested in HC *N* (%) of providers referring patients to HC intervention
**Implementation:** The fidelity/consistency of the delivery of an intervention and/or its individual components, in addition to any adaptations to the intervention and their implications	The extent to which one or multiple EIM components are integrated in the healthcare setting as intended; can describe any adaptations to the workflow and its implications on outcomes	Adaptations to clinical workflow on Reach, Effectiveness, and Adoption metrics
**Maintenance:** The extent to which an intervention becomes part of an organization’s routine practice or policy, or, at the individual level, the persistent effects (positive or negative) of an intervention	The extent to which components of EIM are institutionalized into an organization’s routine care; at individual level, the maintenance of PA and other outcomes (e.g. biomarkers, chronic disease burden, health care utilization)	Variability in Reach, Effectiveness, and Adoption metrics in last 6 and 12 months of evaluation period

### Data analysis

Monthly clinic-level data were extracted from Epic and cleaned in RStudio. Specifically, the monthly number of adult patients who were: (i) screened for inactivity; (ii) identified as insufficiently active; (iii) interested in HC; and (iv) referred to HC were ascertained. The monthly number of providers seeing patients who requested a health coach and the number of providers who referred patients to HC programs were also extracted. The number of patients who completed the online HC request form and connected with a HC program was gathered from an external online survey (Qualtrics LLC, Provo, UT). RE-AIM outcomes were described over the 21 months that aligned with the recruitment period for our group’s recent 12-week HC intervention [22, 25]. Based on the distribution and skewness of the data, means (SD) and medians (min-max) are reported to describe measures of central tendency and dispersion.

To examine RE-AIM outcomes pre- and post-implementation of workflow modifications, months were aggregated into: (i) pre-modification and (ii) post-modification months, and absolute and percentage changes in workflow metrics were described. Specifically, to examine changes pre- and post-implementation of the modification to the health coach interest question on patient interest in HC, months 1–4 (pre), and 5–21 (post) were compared. Conversely, to describe changes pre- and post-language modification on the number and proportion of patients referred to and connected with HC programs, months 1–4 (pre), and 5–9 (post) were compared. Only months 5–9 were included in this “post-modification” period because the second modification requiring providers to address patient HC referral requests, which was implemented in month 10, confounds the possible effects of the first modification on HC program referral and connection. Similarly, changes to the number and proportion of patients referred to and connected with HC programs pre- and post-implementation of the second modification were compared between months 5–9 (pre) and 10–21 (post).

To quantify the maintenance and stability of this clinical workflow, we reported estimates of central tendency and dispersion of Reach, Effectiveness, and Adoption outcomes during the final 6 and 12 months of the evaluation period. As all outcomes during these periods were normally distributed upon visual inspection of histograms and Q-Q plots, we calculated means, standard deviations (SDs), and coefficients of variation (CVs). CVs were calculated by dividing each outcome’s SD by the mean * 100 (CV = [SD/mean] * 100), with higher values indicating less stability and maintenance. As this retrospective evaluation of this clinical workflow was descriptive in nature, we did not have any pre-specified hypotheses, and no null-hypothesis significance testing was conducted.

## Results

### Reach

**Figure 2:**
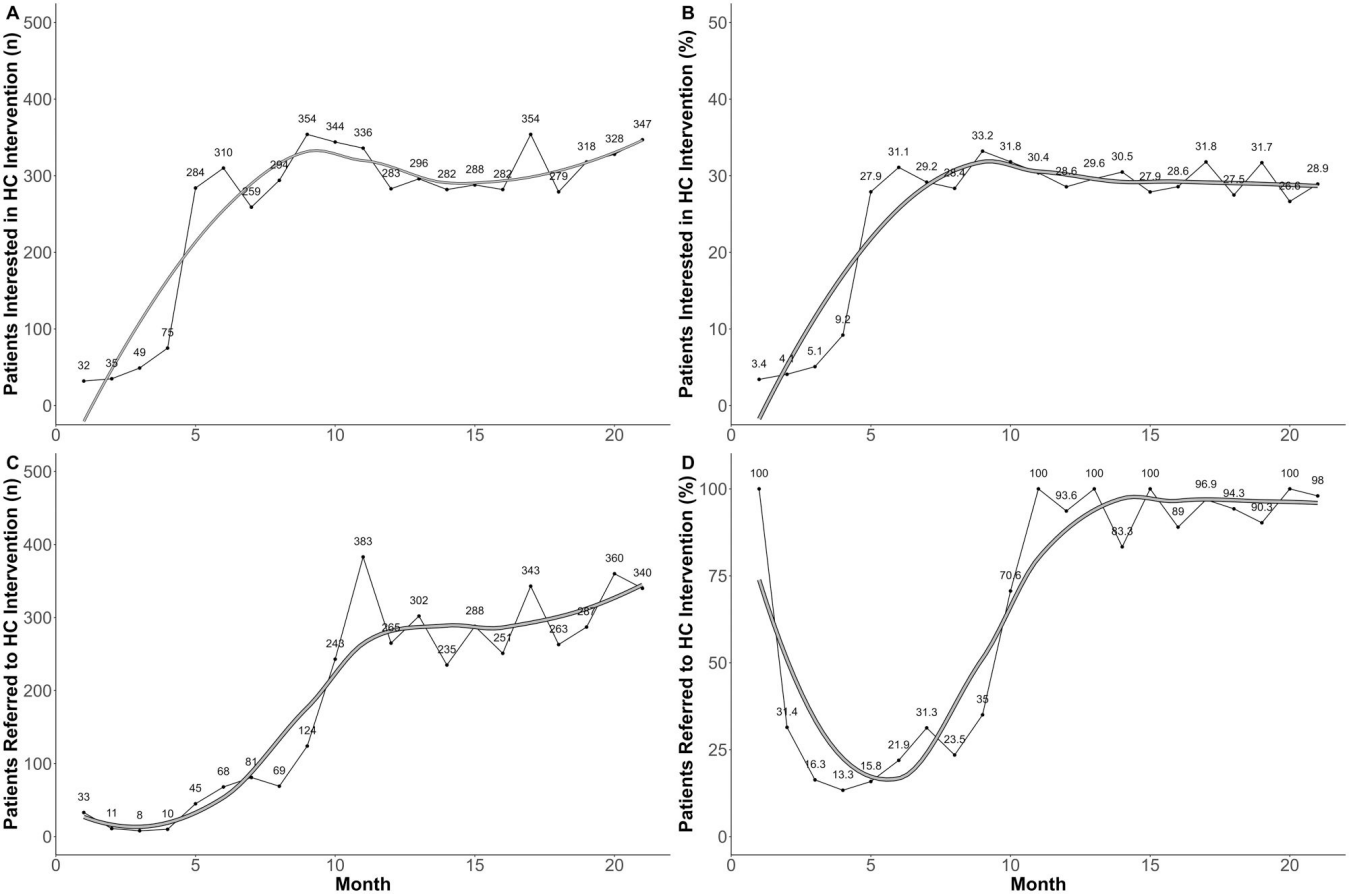
Number of patients interested (A) and referred (C), and proportion of patients interested (B) and referred (D) to HC.

Table 2 and Fig. 2 describes the Reach, Effectiveness, and Adoption of this clinical workflow overall and by clinic. Overall, a mean (SD) of 1771 (153) patients were screened for physical inactivity monthly during annual physical exams. A mean (SD) of 1013 (102), or 57.1% of screened patients, were identified as being insufficiently active. A monthly median (min-max) of 288 (32–354), or 29% of insufficiently active patients, were interested in meeting with a health coach. A median (min-max) of 243 (8–383) insufficiently active patients, or 89% of HC-interested patients, were referred to HC each month.

**Table 2:** Reach, effectiveness, and adoption metrics, overall and by individual clinic.

	All clinics	Clinic 1	Clinic 2	Clinic 3	Clinic 4	Clinic 5	Clinic 6
**Reach**							
Patients seen, *n* (SD)	1699 (167)	126 (25)	443 (45)	395 (52)	144 (30)	189 (28)	412 (47)
Patients screened for inactivity, *n* (SD)	1771 (153)	133 (27)	444 (37)	412 (49)	153 (35)	197 (28)	432 (48)
Patients screened for inactivity, % (SD)	100 (0)	99.7 (1.1)	99.6 (0.8)	100 (0.2)	99.2 (2.2)	99.5 (1.3)	99.9 (0.4)
Insufficiently active patients, *n* (SD)	1013 (102)	81 (17)	244 (24)	234 (29)	89 (20)	125 (25)	240 (27)
Insufficiently active patients, % (SD)	57.1 (2.5)	61.1 (4.3)	55.1 (3.8)	56.7 (3.6)	58.1 (4.1)	63.3 (5.2)	55.6 (2.2)
Insufficiently active patients requesting HC referral, *n* (min-max)	288 (32–354)	27 (0–32)	72 (1–93)	69 (27–91)	26 (0–34)	29 (0–47)	66 (0–89)
Insufficiently active patients requesting HC referral, % (min-max)	29 (3–33)	32 (0–42)	31 (0–37)	31 (14–37)	27 (0–28)	22 (0–52)	32 (0–100)
Insufficiently active patients referred to HC, *n* (min-max)	243 (8–383)	26 (0–42)	69 (0–113)	61 (5–118)	15 (0–28)	24 (0–52)	32 (0–100)
Insufficiently active patients referred to HC, %, (min-max)	25 (1–35)^a^	29 (0–44)	28 (0–46)	26 (3–48)	20 (0–29)	19 (0–33)	14 (0–37)
	89 (13–100)^b^	96 (0–100)	79 (0–100)	91 (17–100)	67 (0–100)	78 (0–100)	45 (0–100)
**Effectiveness**							
Insufficiently active patients connected with HC, *n*	20 (3–37)	–	–	–	–	–	–
Insufficiently active patients connected with HC, %	1.9 (0.2–3.4)^c^	-	-	-	-	-	-
	6.8 (2.8–28.1)^d^						
	9.8 (3.1–45.5)^e^						
**Adoption**							
Providers with insufficiently active patients requesting HC referral, *n*	72 (15–82)	4 (0–6)	30 (0–40)	17 (13–28)	7 (0–9)	8 (0–15)	16 (0–19)
Providers referring insufficiently active patients to HC, *n*	61 (3–79)	3 (0–5)	24 (0–36)	14 (3–19)	2 (0–4)	5 (0–10)	9 (0–16)
Providers referring insufficiently active patients to HC, %	86 (15–100)	75 (0–100)	81 (0–100)	62 (18–100)	38 (0–100)	67 (0–100)	53 (0–100)

SD = standard deviation; HC = health coaching.

a Median proportion of patients referred relative to all insufficiently active patients.

b Median proportion of patients referred relative to all HC-interested patients.

c Median proportion of patients connected relative to all insufficiently active patients.

d Median proportion of patients connected relative to all interested patients.

e Median proportion of patients connected relative to all referred patients.

### Effectiveness

**Figure 3:**
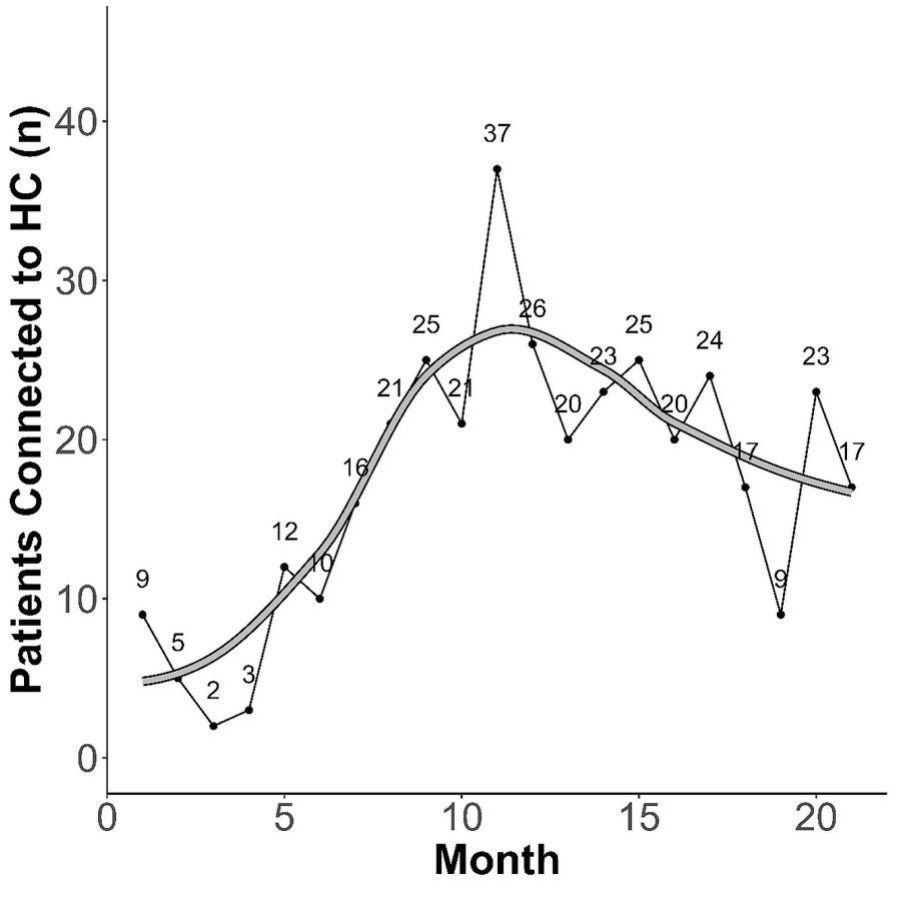
Number of patients connected to HC over 21-month evaluation period.

A monthly median (min-max) of 20 (3–37) referred patients were connected with HC (Fig. 3). This represented 1.9% (0.2%–3.4%), 6.8% (2.8%–28.1%), and 9.8% (3.1%–45.5%) of patients who were insufficiently active, interested in HC, and referred to HC, respectively.

### Adoption

Monthly, a median of 72 providers treated patients requesting to meet with a health coach. Of these providers, 61 (86%) referred patients to HC each month.

### Implementation

**Figure 4:**
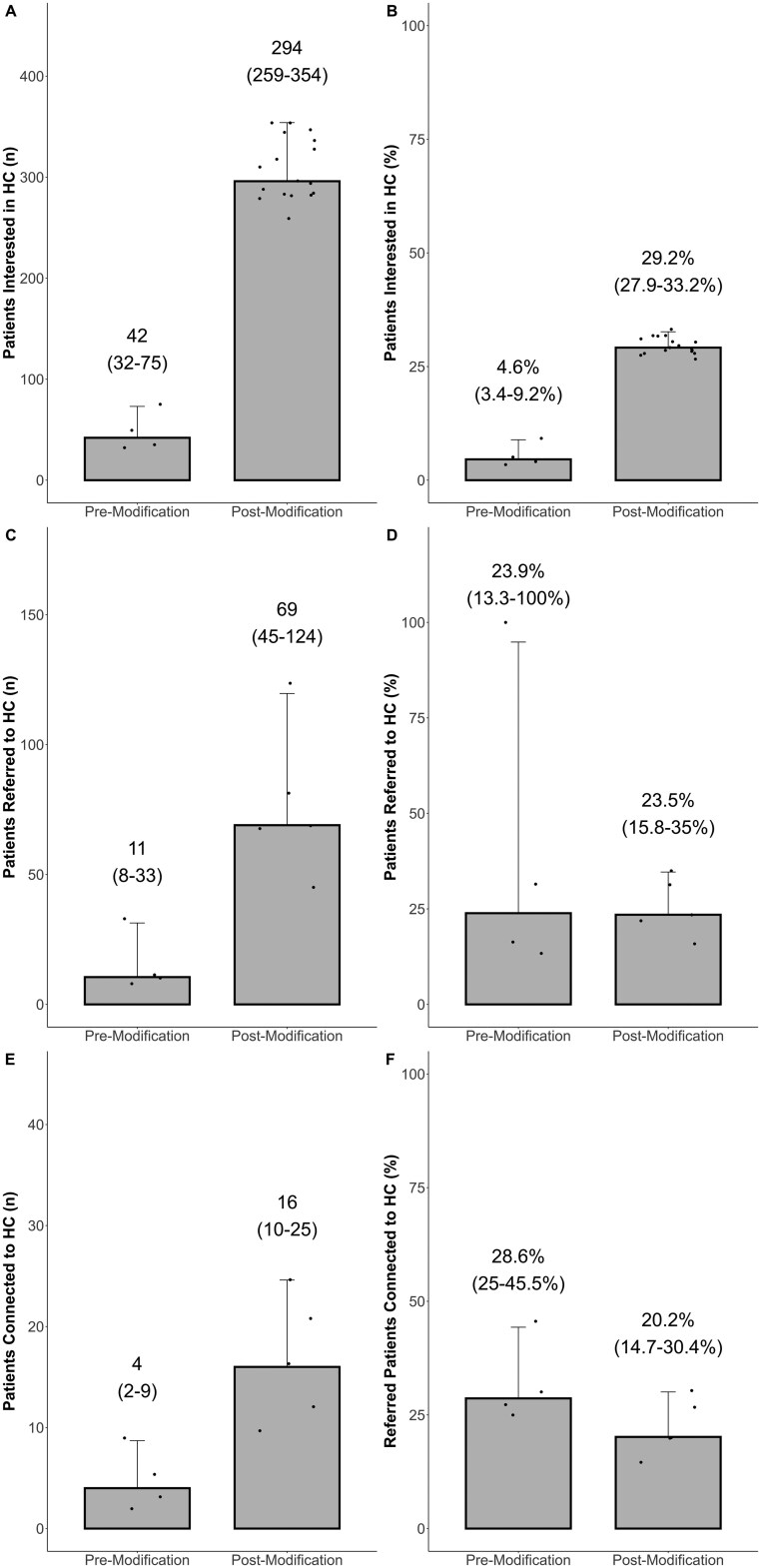
Reach (A-D) and effectiveness (E and F) of clinical workflow pre- and post-health coach interest question language modification.

Figure 4 illustrates changes in Reach and Effectiveness outcomes pre- and post-modifying the language to the health coach interest question. Pre- to post-modification, the monthly median number of patients interested in HC increased from 42 to 294, with the median proportion of patients interested increasing from 4.6% to 29.1% (Fig. 4A and B). Moreover, the median number of patients referred to HC each month increased from 11 to 69, while the median proportion of patients interested in HC who got referred was unchanged (Fig. 4C and D). Finally, the monthly median number of patients connected with HC increased from 4 to 16, while the median proportion of referred patients who were connected to HC decreased from 28.6% to 20.2% (Fig. 4E and F).

**Figure 5:**
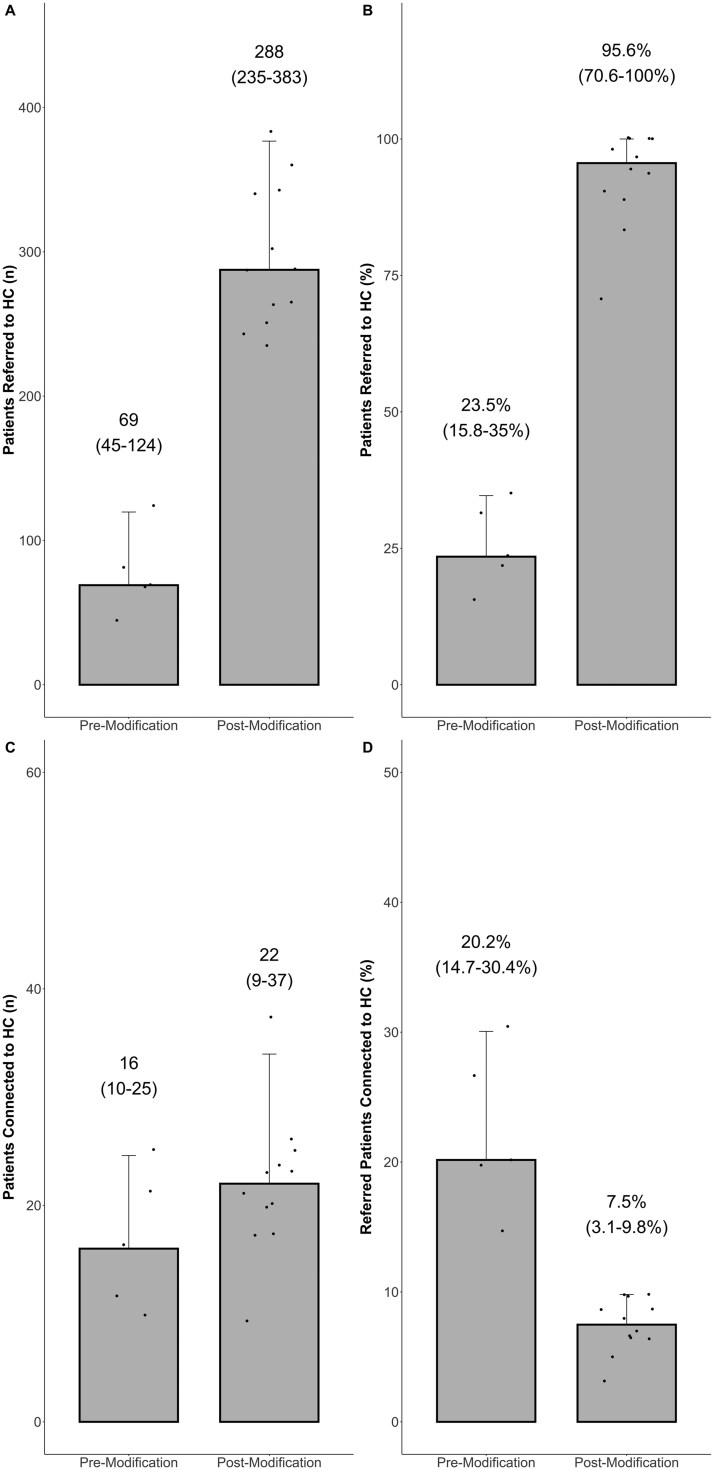
Reach (A and B) and effectiveness (C and D) of clinical workflow pre- and post-requiring providers to address patient request for HC referral.

The changes in ‘Effectiveness’ outcomes from pre- to post-implementation of the modification requiring providers to address the OPA alerting them of their patients’ interest in meeting with a health coach are shown in Fig. 5. The median number of patients referred to HC each month increased from 69 to 288, while the proportion of patients referred increased from 23.5% to 95.6%. Moreover, the monthly median number of patients connected to HC increased from 16 to 22. Pre-modification, the proportion of all insufficiently active patients, HC-interested patients, and referred patients connected to HC was 1.8%, 6.2%, and 20.2%, respectively. Post-modification, the proportion of all insufficiently active patients, HC-interested patients, and referred patients connected to HC was 2%, 6.9%, and 7.5%, respectively.

### Maintenance

Estimates of central tendency and dispersion of workflow metrics over the final 6 and 12 months of the evaluation period are shown in Table 3. During the final 6 and 12 months, most ‘Reach’ and ‘Adoption’ metrics demonstrated low variability (CVs = 0%–16.6%). In contrast, ‘Effectiveness’ outcomes demonstrated high variability. Specifically, high variability was exhibited in the number and proportion of patients connected to HC (CVs = 27.6%–30.4%).

**Table 3:** Maintenance of reach, effectiveness, and adoption outcomes during the last 6 and 12 months of the evaluation period.

	6 months				12 months		
Outcome	Mean	SD	CV		Mean	SD	CV
**Reach**							
Patients seen, *n*	1812	153	8.4%	1784		131	7.3%
Patients screened for inactivity, *n*	1876	161	8.6%	1848		127	6.8%
Patients screened for inactivity, %	100%	0%	0.0%	100%		0%	0.0%
Insufficiently active patients, *n*	1092	106	9.7%	1057		91	8.7%
Insufficiently active patients, %	58.2%	2.8%	4.8%	57.2%		2.5%	4.4%
Insufficiently active patients requesting HC referral, *n*	318	32	10%	311		29	9.4%
Insufficiently active patients requesting HC referral, %	29.2%	2.1%	7.3%	29.5%		1.8%	6.0%
Insufficiently active patients referred to HC, *n*	307	46	15.0%	297		49	16.6%
Insufficiently active patients referred to HC, %	28.1%^a^	2.0%	7.3%	28%		3.1%	11.2%
	94.7%^b^	4.4%	4.6%	93%		8.8%	9.5%
**Effectiveness**							
Insufficiently active patients connected with HC, *n*	18.3	5.4	29.6%	21.8		6.6	30.4%
Insufficiently active patients connected with HC, %	1.7%^c^	0.5%	27.6%	2.1%		0.6%	30%
	5.8%^d^	1.7%	28.7%	7.1%		2.1%	29.8%
	6.0%^e^	1.7%	28.4%	7.4%		2.1%	27.7%
**Adoption**							
Providers with insufficiently active patients requesting HC referral, *n*	73.1	5.9	8.2%	73.8		5.8	7.9%
Providers referring insufficiently active patients to HC, *n*	69.7	6.7	9.5%	68.2		6.2	9.1%
Providers referring insufficiently active patients to HC, %	95.7%	3.6%	3.7%	91.6%		5.7%	6.1%

SD = standard deviation; CV = coefficient of variation; HC = health coaching.

a Proportion of patients referred relative to all insufficiently active patients.

b Proportion of patients referred relative to all HC**-**interested patients.

c Proportion of patients connected relative to all insufficiently active patients.

d Proportion of patients connected relative to all HC-interested patients.

e Proportion of patients connected relative to all referred patients.

## Discussion

The aim of this study was to systematically describe the initial implementation of a clinical workflow that identifies, refers, and connects insufficiently active patients to HC. Nearly 60% of adult patients did not meet aerobic PA guidelines, and about one-third of these patients were interested in meeting with a health coach. Most patients who were interested in HC were referred by their provider, yet less than 10% ultimately connected with a health coach, highlighting a clear gap between patient intentions and connection with HC. Future work should identify and address the multi-level barriers limiting patients’ connection to supportive behavior change resources.

While the inactivity estimates in this study are higher than population-level estimates [12], they align well with estimates in other primary care clinics [26–28]. The discrepancy between clinic and population-level estimates may reflect the relatively poorer health of primary care patients relative to the general U.S. population. Indeed, middle-aged and older adults, who generally have higher NCD burden compared to their younger counterparts [29], may visit healthcare providers more frequently. Furthermore, it is well-established that patients with greater NCD burden, compared to lower burden, engage in less PA [30]. Given the consistent link between PA and reduced risk of NCDs [2, 4, 6–9, 11], our findings reinforce the population health importance of promoting PA within primary care.

Despite high levels of inactivity among patients in this study, few patients expressed interest in meeting with a health coach. This finding contrasts with Falskog and colleagues who reported that approximately 50% of general practice patients want help from their healthcare provider(s) to increase their PA [31]. However, the role of health coaches within the U.S. healthcare system is not well elucidated and patients have varied perceptions of HC [32], which may partially explain these divergent findings. Indeed, there are no federal regulations for HC, and recent evidence suggests ∼90% of practicing U.S. health coaches are not National Board-Certified Health & Wellness Coaches [33]. This may negatively impact patient perceptions and attitudes toward HC. Akin to our findings, Dayao and colleagues described the effectiveness of their workflow connecting insufficiently active U.S. primary care patients to a phone-based HC program and found only 7% of patients self-referred to HC [26]. This is unfortunate, as HC can elicit positive behavioral changes, improve health and disease biomarkers, and increase trust between patients and providers [34–36]. Collectively, this highlights a need to clarify the role of HC in patient care across multiple key stakeholders (i.e. patients, providers, healthcare administrators, and policy makers). Doing so may result in a more systematic and uniform implementation of HC within the U.S. healthcare system, improving patient care and health outcomes.

The findings from this study provide indirect evidence that elucidating the role of a health coach in primary care may increase the reach of HC. Specifically, marked increases in patient HC interest were observed after clinics modified the language of the HC interest question, implemented, in part, to clarify the role of a health coach. It is important to note, however, that this modification coincided with the full implementation of this workflow in four of six evaluated clinics (Supplementary Figs S2 and S3), likely confounding the apparent effects of this modification. Nevertheless, these findings may suggest some degree of plasticity in patient perceptions and attitudes toward HC and may warrant the development and evaluation of both patient and provider education-based implementation strategies to augment the reach and overall impact of HC.

Despite all clinics fully implementing this workflow and clarifying the role of HC by month five, the proportion of patients referred to HC when requested was persistently low. Specifically, across months 1–9, less than a quarter of patients interested in HC received a referral from their provider. While the explanation for this is beyond the scope of this study, it could be that providers were largely unaware of their patients’ interest in meeting with a health coach. Indeed, when clinical leadership implemented a modification requiring providers to address the OPA clinical decision support tool that alerted providers of their patients’ interest in connecting with a HC, patient referrals increased four-fold. Therefore, it seems that when (i) providers are aware of their patients’ interest in receiving a referral and (ii) the referral process is systematically built into existing workflows, providers are supportive and likely to refer. This finding is corroborated by the robust literature highlighting providers’ positive perceptions toward promoting their patients’ PA [37]. While providers are often limited by time, financial incentives, expertise and confidence in promoting PA, these data highlight the importance of developing and refining healthcare-based PA promotion strategies that place minimal burden on providers. Accordingly, future work should explore novel approaches (e.g. automated eReferrals) to refer and connect insufficiently active patients to PA resources from the healthcare setting [38].

While approximately 300 patients were interested in connecting with HC each month, few patients ultimately connected, highlighting a discrepancy between patient intentions and actual connection to supportive PA resources. This phenomenon, termed the “intention-behavior gap”, is well articulated within the context of health behavior change and PA behavior change, in particular [39]. It is estimated that intentions to increase PA translate to increased PA only half the time [39], suggesting other behavior change constructs explain changes in PA. With respect to this current study, intention to connect with a health coach—demonstrated by patient interest in connecting with one—translated to actual connection to HC <10% of the time. We recently reported the preliminary efficacy of our 12-week HC intervention in patients at elevated risk for cardiovascular disease who were referred from this current workflow [25]. Among patients not meeting aerobic PA guidelines at baseline, we observed small-moderate increases in daily steps (*d *= 0.50) and moderate-to-vigorous-intensity PA (*d *= 0.38) pre- to post-intervention. Interestingly, while patients seemed to improve their behavioral skills regulation, health habits, and identity, patients did not increase their attitudes or perceived capability and opportunity—key constructs in the early stages of behavior change [40]. It is plausible that relatively high baseline levels of these latter constructs serve as “pre-requisites” to connect with a health coach, limiting the reach and overall impact of this workflow to patients with the adequate resources (e.g. time, physical and financial access to health-promoting PA facilities and spaces, social support) to connect to offered support. In partial support of this, patients completing our intervention were highly educated and were most often married, which is consistent with evidence suggesting patients of high socioeconomic status are over-represented in health behavior change interventions [41, 42].

Providing additional evidence for differential reach and effectiveness of this workflow across sociodemographic characteristics, patients treated in the sole rural clinic (Clinic 5) were the most likely to be insufficiently active and were the least likely to express interest in meeting with a health coach (Table 2; Supplementary Figs S1 and S2). It is well-appreciated that rural adults engage in less PA compared to non-rural adults [43, 44], likely due to numerous individual, interpersonal, and sociopolitical factors [45–47]. It therefore seems plausible that external factors might also explain low connection rates to HC, especially within clinics serving patients who are subjected to health inequities. Future evaluations of physical inactivity screening and referral workflows should describe the extent to which they may be a mechanism to ameliorate or perpetuate health disparities and explore and implement strategies to ensure their equitability.

While increasing the number and proportion of patients connected to effective, evidence-based resources should remain a clear goal for all clinical workflows, we have shown that provider engagement can be induced and sustained with clinic leadership-led changes and support (Table 3; Supplementary Fig. S4). Care should be taken, however, when implementing system-level changes, as these modifications may result in unintended negative consequences. Specific to this workflow, implementing a clinical decision support tool that required providers to address their patients’ interest in HC (i.e. an OPA) resulted in nearly all interested patients being referred. At the same time, however, reductions of nearly 70% were observed in the proportion of referred patients connected to HC (Fig. 5D). While the explanation for this is not entirely clear, it seems plausible that pre-modification referring providers were more likely to discuss what HC is and/or what a patient could expect to gain by meeting with a health coach, compared to providers referring out of necessity, post-modification. In other words, requiring providers to address each patients’ referral request increased the volume of referrals but may have diluted their quality. This may also explain the relatively high variability in ‘Effectiveness’ metrics (Table 3). These data highlight the need to better understand patient-provider interactions within this workflow, as modifications may unintentionally reduce impact. Future work could explore evaluating these interactions by extracting and qualitatively analyzing provider notes and/or by documenting the use of SmartPhrases or EPIC dot phrases within the EMR [26]. These findings also illustrate the importance of collaborating with both patient and provider stakeholders in the development and implementation of modifications to clinical workflows to ensure their high relevance, acceptability, and overall impact.

This study has notable strengths and limitations. Strengths include the systematic use of a validated evaluation framework and ecological validity. By applying the RE-AIM framework, we demonstrate a model for operationalizing and evaluating clinical workflows aimed at connecting insufficiently active patients to supportive resources. We were also able to explore changes in workflow metrics pre- and post-implementation modifications and highlight their possible implications. This enables future investigators and clinical decision-makers to implement and evaluate context-specific modifications to improve the rate at which workflows elicit meaningful improvements in patient care and health. Limitations to this study include reliance on aggregate, clinic-level data, which prevented describing and comparing patient characteristics across the workflow. This limits our ability to determine which patients the workflow is and is not reaching to tailor future, equitable implementation strategies. The use of this aggregate data also complicates interpretation. For example, in multiple months of this evaluation, the number of patients referred slightly exceeded the number of patients interested in referral. While the explanation of this is not entirely clear, it could be that some patients did not initially express interest in a referral but, after discussion with their provider, were referred. While this does not materially influence the interpretation of our findings, future work should clarify the source(s) of discrepancy within these data for more precise and robust evaluations.

Clinic leadership also implemented two modifications to the workflow during the evaluation period, which we try to estimate the impacts of using simple pre-post descriptive analyses. Caution should be used in interpreting these “effects”, however, as it cannot be concluded that these changes represent effects of these modifications, per se. Rather, maturation of the workflow across clinics and providers, residual and latent effects of previous modifications, undocumented modifications within individual clinics, and/or seasonality effects [48] may confound the true effect sizes of these modifications. Future work, including the inclusion of non-intervention comparison clusters (e.g. clinics, providers), should clarify how best to implement, analyze, and interpret the effects of modifications to ensure methodological rigor. Finally, while this evaluation identified potential targets for future modifications, it does not specify which strategies should be selected and how they should be implemented. Qualitative work with patients and clinical stakeholders is needed to identify the contextual factors currently hamstringing the workflow’s impact.

## Conclusion

This study described the initial implementation of a clinical workflow designed to identify, refer, and connect insufficiently active patients to behavior change support (i.e. health coaching). Our findings demonstrate this workflow can identify insufficiently active patients seeking behavioral support and provides patients with referrals to supportive resources. Our findings also identified an intention-behavior gap, however, with many patients interested in getting connected with a health coach but few actually getting connected. Future work should better understand the contextual, multi-level barriers preventing patients from connecting with these resources, to develop a scalable, equitable, and sustainable approach to augment patient care, health, and quality of life. Doing so may mitigate the individual and social burdens associated with inactivity-attributable NCDs.

## Funding sources

The authors received no specific funding for this work.

## Supplementary Material

ibag036_Supplementary_Data

## Data Availability

De-identified data from this study will be made available by emailing the corresponding author(s).

## Welfare of animals

This article does not contain any studies with animals.
