# Supplementary material for: Using the RE-AIM framework to evaluate the implementation of a clinical workflow designed to identify, refer, and connect insufficiently active patients to health coaching
Source: Transl Behav Med. 2026 Jul 8;16(1):ibag036. doi: 10.1093/tbm/ibag036 (PMC13344864; doi:10.1093/tbm/ibag036)

**Using the RE-AIM Framework to Evaluate the Implementation of a Clinical Workflow Designed to Identify, Refer, and Connect Insufficiently Active Patients to Health Coaching: Supplementary Figures**

**Table of Contents**

[**Supplementary Figure 1: Number and proportion of insufficiently active patients, by clinic.** 2](#_Toc225249900)

[**Supplementary Figure 2: Number and proportion of insufficiently active patients interested in HC, by clinic.** 3](#_Toc225249901)

[**Supplementary Figure 3: Number and proportion of insufficiently active patients referred to HC, by clinic.** 4](#_Toc225249902)

[**Supplementary Figure 4: Number and proportion of providers referring to HC, by clinic.** 5](#_Toc225249903)

# **Supplementary Figure 1: Number and proportion of insufficiently active patients, by clinic.**

**
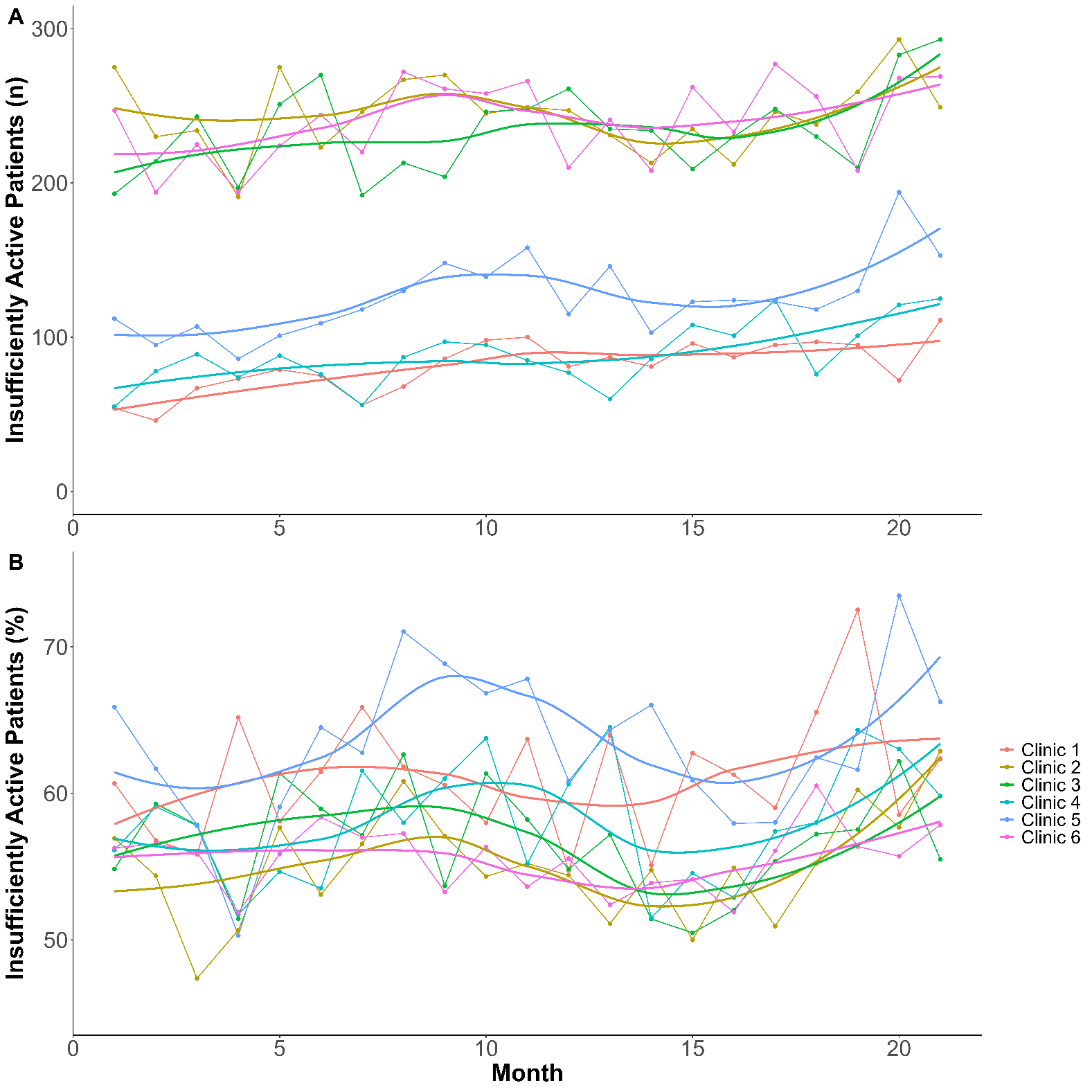
**

# **Supplementary Figure 2: Number and proportion of insufficiently active patients interested in HC, by clinic.**


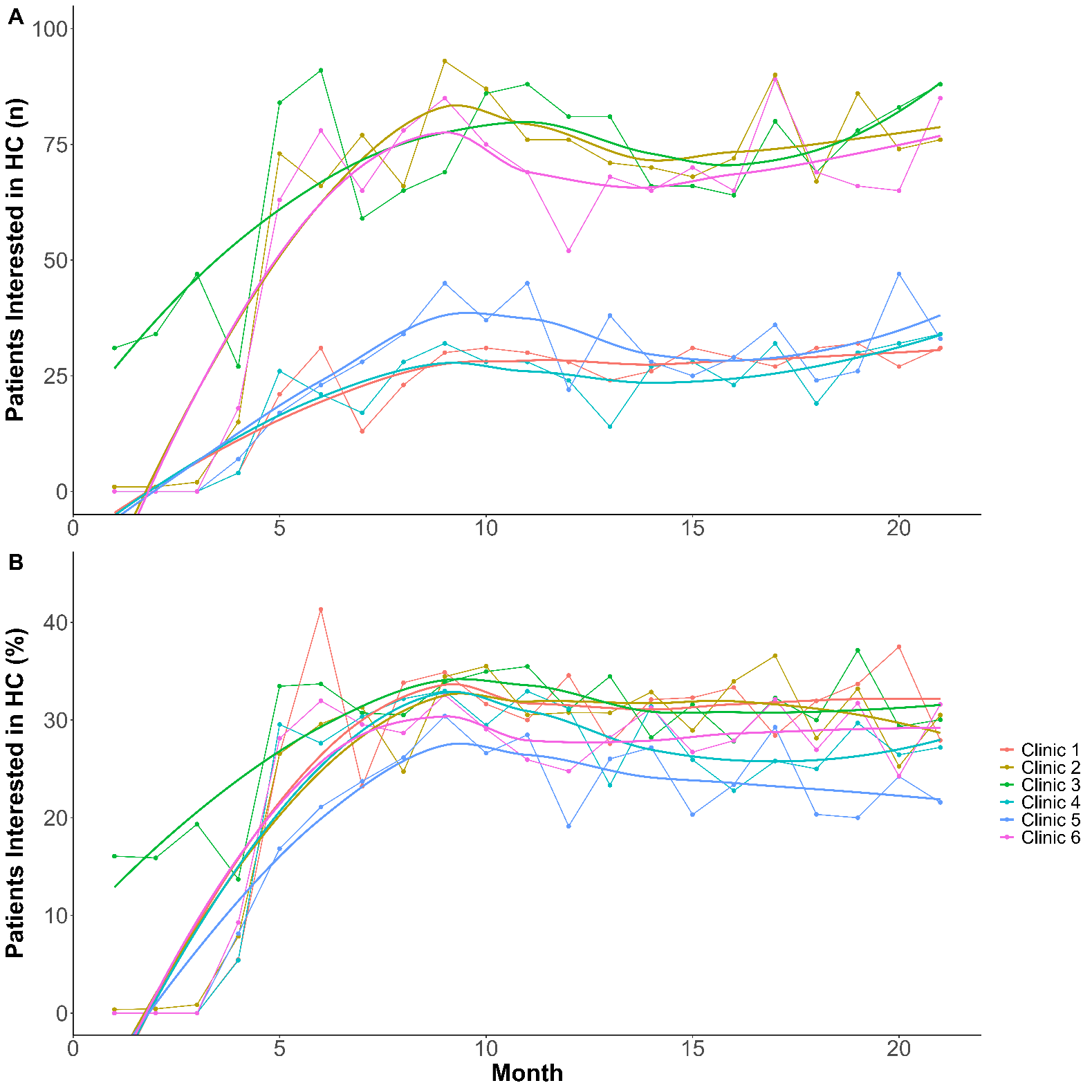


# **Supplementary Figure 3: Number and proportion of insufficiently active patients referred to HC, by clinic.**


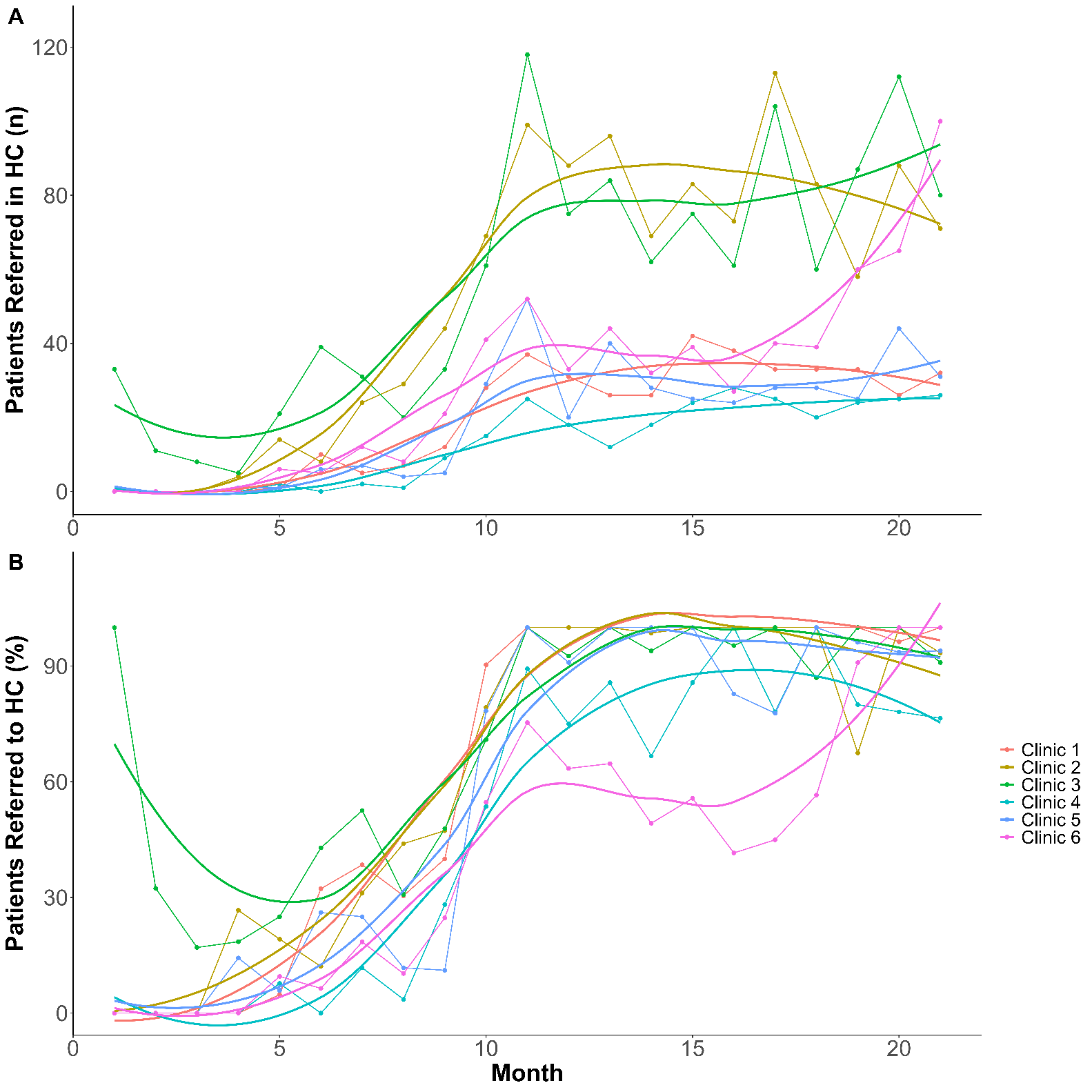


# **Supplementary Figure 4: Number and proportion of providers referring to HC, by clinic.**


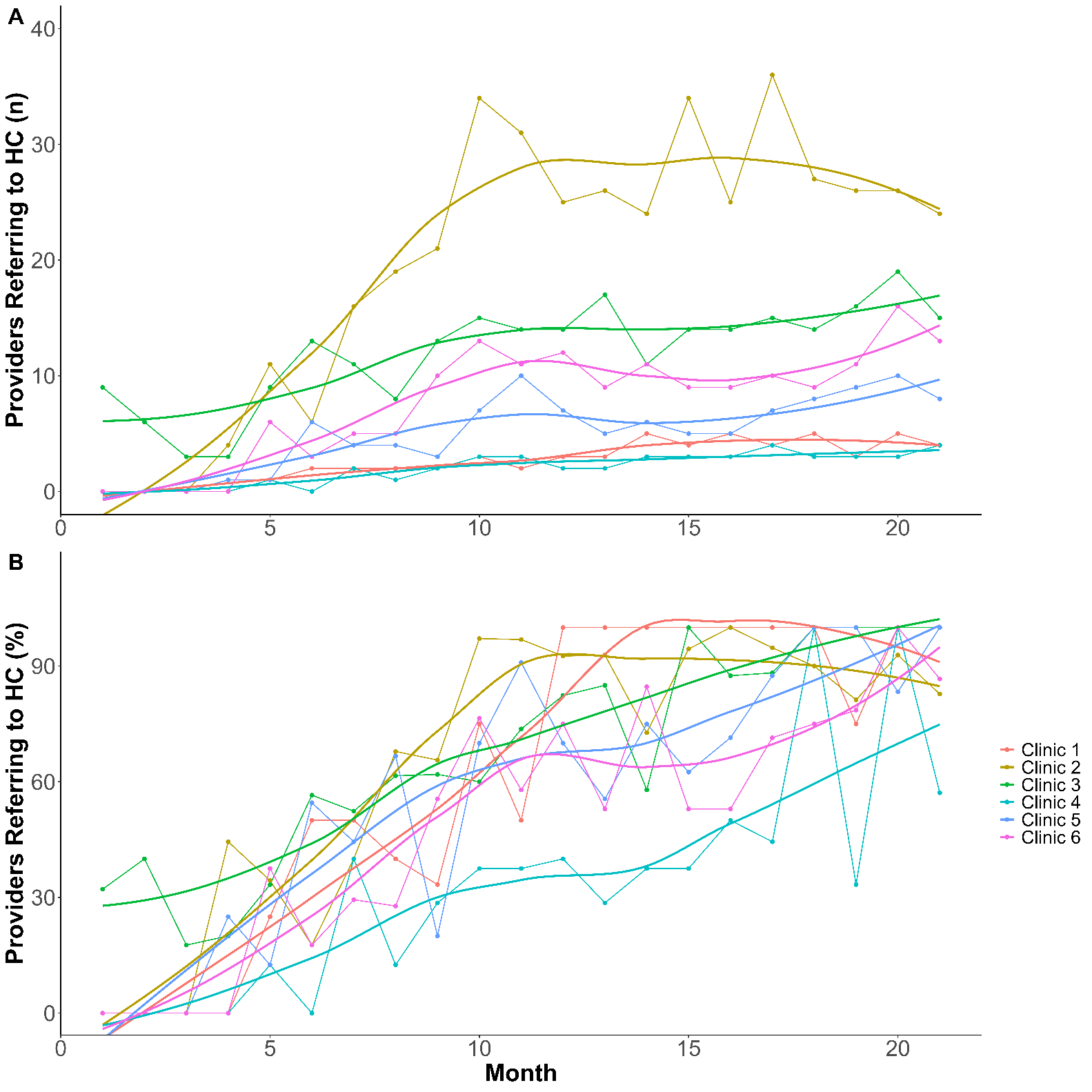

Supplement: ibag036_Supplementary_Data [file ibag036_supplementary_data.docx]
